# Agreement Between Visual Assessment of Urinary Stones Morphology During Endo-Urological Procedure and Fourier Transform Infrared Spectroscopy-based Composition Analysis: An Observational Study

**DOI:** 10.31729/jnma.v63i290.9203

**Published:** 2025-09-01

**Authors:** Nirmal Rasali, Anil Shrestha, Parash Mani Shrestha, Robin Bahadur Basnet, Baikuntha Adhikari, Arvind Kumar Shah, Udita Mishra, Rudra Dahal, Bir Bahadur Thapa

**Affiliations:** 1 Department of Urology, National Academy of Medical Sciences, Bir Hospital, Mahaboudha, Kathmandu, Nepal

**Keywords:** *stone analysis*, *urolithiasis*, *visual assessment*

## Abstract

**Introduction:**

To aim of study was to predict the success rate of the stone composition by visual assessment during endourological procedures, using Fourier transform infrared spectroscopy as a reference standard.

**Methods:**

Prospective observational study showed, 150 patients undergoing endourological procedures for urolithiasis between June 2024 and July 2025 were analyzed. The senior consultant, the junior consultant, and the residents visually assessed the stone morpho-constitution (according to Daudon classification) during the endoscopic procedures. Stone samples were obtained which were analyzed using Fourier transform infrared spectroscopy, and the results were correlated with the visual assessment done by the surgeon.

**Results:**

Stone analysis by Fourier transform infrared spectroscopy showed 28 (18.67%) of the stones had only one morphological type (pure stones), while 122 (81.33%) had more than one composition of stones (mixed stones). The accurate predictions for pure stones as Uric acid stones and Protein stones, were 43 (84.31%) and 5 (83.33%), respectively, whereas for Calcium Oxalate Monohydrate + Calcium Oxalate Dihydrate (mixed stones) was 126 (79.25%). The overall correct prediction was obtained in 64.44 %. The correct prediction was highest by the senior consultant 110 (73.33%), followed by the junior consultant 97 (64.67%), and the residents 83 (55.33%), respectively.

**Conclusions:**

The overall agreement between visual assessment of urinary stone morphology during endo-urological procedures and Fourier transform infrared spectroscopy-based composition analysis in this study was moderate, with a Cohen’s kappa value of 0.62.

## INTRODUCTION

Urolithiasis affects 2 to 20.00% of the global population, with recurrence rates approaching 50.00% within five years.^1,2^ Understanding stone composition is at the core of effective management, and for recurrence preventive strategies.^3^ While formal stone analysis remains the standard of care for determining composition, immediate visual assessment during endoscopic procedures could provide valuable intra-operative information to guide treatment decisions and patient counseling.^4,5^

Previous studies evaluating endoscopic stone recognition have utilized static images rather than real-time assessment.^6^ These approaches may not represent the entire stone appearance. Further, limited data exist on the influence of surgical experience on visual assessment accuracy, particularly in resource constrained settings where stone analysis may not always be available.

This study aimed to evaluate the correct prediction of visual stone composition by urologists during endoscopic procedures, using Fourier transform infrared spectroscopy (FTIR) as a reference standard.

## METHODS

This is an observational, prospective study. Patients undergoing endourological procedures for nephrolithiasis were the study population. The study was carried out in the Department of Urology, National Academy of Medical Sciences (NAMS), Bir Hospital, Nepal between June 2024 and July 2025. Ethical approval was obtained from the IRB, NAMS, and informed written consent was taken from all the patients (IRB reference number- 99/2081/82). Patients undergoing Percutaneous Nephrolithotomy (PCNL) or Retrograde Intra Renal Surgery (RIRS) for urolithiasis and stone analysis done were included in the study. Patients with active urinary tract infection, inability to inspect the stone, and patients in whom retrieval of the stone was not possible and stone analysis was not performed were excluded from the study.

The sample size was calculated for a hypothesis test of interrater agreement (Visual assessment vs. FTIR analysis) measured by Cohen’s Kappa and the calculated sample size was 150. The sample size for this study was calculated by assuming a null hypothesis of substantial agreement (κ_0_ = 0.6) against the alternative hypothesis of near-perfect agreement (κ_1_ = 0.8).^4^ This reflects the study’s aim to determine if visual inspection can achieve a very high level of diagnostic accuracy. A twosided significance level (α) of 0.05 and a power (1 - β) of 80% were chosen. The sampling technique was non-probability consecutive sampling who met the inclusion criteria during the study period.

Demographic parameters, including history and physical examination, were recorded. Stone volume (mm^3^), density (Hounsfield Unit, HU), and location were determined preoperatively using Non-contrast Computed Tomography of the kidney, ureter, and bladder. RIRS was performed by a 7.5 Fr disposable flexible digital ureteroscope (Indoscope Sleek™, Bioradmedisys, India), and PCNL was performed by a 12 Fr Nephroscope (Karl Storz™ Tuttlingen, Germany). Surgeons (senior consultant, junior consultant, and residents) were asked to assess stone composition after stone visualisation endoscopically, as per Daudon Classification into seven groups with subgroups.^7-9^ The surgeons were asked to assess the stone morphology separately and assessors were instructed not to discuss the stone appearance or view others’ entries until all three submissions were complete. Visual assessment was based on the initial impression of stone morphology and during various phases of lithotripsy and the final response was recorded after the procedure and the response was collected in the proforma designed for the study. The stone fragments were retrieved using a basket or suction sheath in RIRS and by irrigation in PCNL, and all the stones were sent for analysis using the Fourier transform infrared spectroscopy (FTIR) technique. The correct prediction of the stone morphology was evaluated by comparing the visual assessment prediction with FTIR stone analysis and the correct prediction rate was calculated by prediction via Visual Assessment by Surgeons / FTIR x 100. Urologists were categorized into senior consultants (>5 years of experience), junior consultants (1-5 years of experience), and Urology residents. Each patient had three visual assessment of urinary calculus by Senior Consultant, Junior Consultant and Resident with a total response of 450. The gold standard of stone analysis was FTIR for correct prediction. The stone was considered pure stone if it had a single composition or more than 90 % of the stone’s composition, and mixed stones if with two or more compositions.^10,11^ In case of mixed stone, assessment of the stone morphology was considered correct when two components of the stone’s composition were predicted correctly.^16^ The pure stones were predicted more correctly than the mixed stones.

The data were analyzed using descriptive and comparative methods. Continuous variables, such as age, BMI (Body Mass Index), stone volume (π/6 × Length in mm × Width in mm × Height in mm), and stone density, were expressed as mean±standard deviation (SD) and median, with ranges while categorical variables, including sex, stone laterality, and procedure type, were reported as frequencies and percentages, n (%). The correct prediction of stone type was calculated as the percentage of correct identifications relative to the total cases. Subgroup analysis were performed to compare correct prediction rates between pure and mixed stones and levels of clinical experience (senior consultants, junior consultants, and residents). Statistical analyses were conducted using IBM SPSS Statistics version 25.0 (IBM Corp., Armonk, NY, USA).

## RESULTS

A total of 178 patients underwent endourological procedures for urolithiasis, out of which 150 patients were included in the study. Among the total, 28 patients were excluded (22-stone analysis was not available, 6-bleeding was present, obscuring the vision of the stone). Among them male were 93 (62%)and the female were 57 (38%). The mean age of 41.4±15.2 years and BMI was 24.8±4.2 kg/m^2^ and the stone density was 1099±183 HU (Table 1).

**Table 1:** Characteristics of study population with renal stone (n=150).

Variable / Category	Value
Age (years, mean±SD)	41.4±15.2
**Sex**	
Male	93(62.00)
Female	57(38.00)
BMI (kg/m^2^, mean±SD)	24.8±4.2
Stone Size (mm^3^, median [range])	2305 (IQR:540-20160)
Stone Density (HU, mean±SD)	1099±183
**Stone Side**	
Left	83(55.33)
Right	67(44.67)
**Procedure**	
PCNL	79(52.67)
RIRS	71(47.33)

SD = Standard Deviation; BMI = Body mass Index; PCNL = Percutaneous Nephrolithotomy; RIRS = Retrograde Intrarenal Surgery; HU = Hounsfield Units; IQR = Interquartile Range.

Each case had three responses from the senior consultant, junior consultant, and residents, with a total response of 450 and a composite correction prediction of 290 (64.44%). The correct prediction made by Senior consultant was 110 (73.33%), junior consultant was 97 (64.67%) and resident was 83 (55.33%). The overall inter-rater reliability was 0.62 (Table 2).

**Table 2:** Concordance between visual inspection by surgeons and Fourier transform infrared (n=150 patients; 450 observations).

Surgeons	Stone Type	Total Cases (n)	Correct Predictions n(%)	Overall correct predictions n (%)	Agreement (Cohen’s Kappa)
Senior Consultant(n1=150)	Pure Stone	28	21(75.00)	110(73.33)	SC vs JC 0.61
	Mixed Stones	122	89(72.95)		
Junior Consultant (n2=150)	Pure Stone	28	20(71.43)	97(64.67)	JC vs Resident 0.50
	Mixed Stones	122	77(63.11)		
Residents (n3=150)	Pure Stone	28	16(57.14)	83(55.33)	SC vs Resident 0.51
	Mixed Stones	122	67(54.92)		

**Table 3:** Composition and frequency of urinary calculi (n=150) and correct predictions of different types of stones (n=150 patients; 450 observations).

Stone Type	n(%)	Total Predictions (n)	Correct Predictions n(%)
**Pure Stones**			
Type I - Whewellite (COM)	4(2.67)	9	7(77.78)
Type II - Weddellite (COD)	3(2.00)	7	5(71.43)
Type III - Uric Acid	12(8.00)	51	43(84.31)
Type IIIb - Ammonium Urate	1(0.67)	3	1(33.33)
Type IVa - Phosphate Carbapatite	2(1.33)	6	2(33.33)
Type IVc - Struvite Stones	4(2.67)	9	6(66.67)
Type VI - Protein Stones (Matrix Stones)	2(1.33)	6	5(83.33)
**Mixed Stones**			
COM + COD	79(52.67)	159	126(79.25)
COM + Uric Acid + Ammonium Urate	33(22.00)	170	83(48.82)
COM + Struvite Stones	10(6.67)	30	12(40.00)

COM = Calcium Oxalate Monohydrate; COD = Calcium Oxalate Dihydrate.

As per FTIR the pure stone was 28 (18.67%) and the mixed stone was 122 (81.33%). By FTIR stone analysis, the calcium oxalate stones were present in 129(86.00%) of the cases, Uric acid stones in 12 (8.00 %), and Struvite stones in 4 (2.60 %) of the cases. Among pure stones, Type Ilia (uric acid) stones had 51 total prediction, of which 43 (84.31%) were correctly predicted with visual assessment by all the surgeons. In mixed stones Calcium Oxalate Monohydrate (COM) + Uric Acid + Ammonium Urate combinations had 170 total predictions and 83 (48.82%) correct predictions with visual assessment by all the surgeons (Table 3).

## DISCUSSION

The inter-observer agreement demon strated moderate concordance, with the higher agreement observed between the senior consultant and junior consultant (κ = 0.61), followed by the senior consultant and resident (κ = 0.51), while the junior consultant and resident showed the lowest agreement (κ = 0.50). The overall agreement among all raters was κ = 0.62, indicating a moderate level of consistency in visual prediction of stone composition.

In this study, the population consisted of 150 patients with urolithiasis who underwent endourological procedures in Nepal, while Randall et al. evaluated a total of 1370 surveys were successfully delivered and 366 were completed, resulting in a response rate of 26.7% in the USA.^4^ However, the methodological focus differed, as this study assessed stones in a real intraoperative setting, whereas Randall et al. relied on image-based evaluations. The exclusion criteria also varied, with this study excluding cases where stone analysis could not be performed due to poor visualization, such as bleeding obscuring the operative field, while Randall et al. did not specify any exclusion criteria.

In the study by Randall et al., the overall stone identification accuracy was 44.00%. COM, Struvite, and COD obtained the most successful identification rates (65.90%, 55.70%, and 52.00%, respectively), whereas the current study identified Uric acid and Protein stones (84.31% and 83.33%, respectively) more successfully. The most frequently misidentified stones were Carbonate phosphate apatite (10.70% and 33.33%) in both Judies.^4^ They concluded that increased accuracy was considered to be due to self-perceived ability to determine composition and number of ureteroscopies per week, while years of experience did not show a positive correlation. However, our study showed an increased prediction rate according to the surgeon’s experience, as the senior surgeon’s prediction rate was 73.33 %, compared to the resident’s, which was 55.33%. So, it can be predicted that the learning curve is present to acquire the morpho-constitutional analysis stone recognition and to apply it practically.

Daudon and Cloutier emphasised the importance of studying urinary stone morphologies as the knowledge of the morpho-chemical composition of the stone is crucial to underhand the mechanism of stone formation and to choose the measures for the prophylaxis of recurrences.^7,12^ In addition, it also helps to plan the strategy of stone treatment. Endoscopic stone recognition can help the surgeon choose the right Laser settings according to the hardness of the stones. The knowledge of the composition of the stone can modify therapeutic choices, as some stones, such as uric acid, can be treated by oral chemolysis, and others, such as struvite stones, show high recurrence, requiring complete clearance of all the fragments.^11,13^ Similarly, matrix stones may require a higher group of intravenous antibiotics to prevent septic complications. Multiple guidelines, including the European Association of Urology guidelines, Wrongly recommend collecting stone material for analysis to classify the stone type.^14^

In this study, the most common composition of the stone was calcium oxalate stones, present in 85.90% of the cases, followed by Uric acid stones, 8.76 %, and then Carbapatite stones, present in 5.33 % of the cases. The most commonly accurately predicted stones were Uric acid stones (84.31%), Protein stones or matrix stones (83.33%), and COM (77.78%), respectively.

Initial study by Sampogna et al. showed an overall accuracy rate of 39% and the current study showed an overall correct prediction of 64.40%.^6^ The prediction was higher in our study because the stone morpho-constitutional analysis was done before and various phases of lithotripsy whereas in the study by Sampogna et al., the study was done with 15 minutes video presentation of eight common stones to the observer. In comparison to our study and by Sampogna et al, COD stones have been correctly detected in 71.43% vs 69.80%, COM in 77.780% vs 41.80%, uric acid in 84.31% vs 33.30% and calcium oxalate + uric acid in 48.82% vs 34.30% and cystine no cases vs 78.10%.^6^ In a study by Bergot et al, 15 junior urologies analysed the morphology of nine stone pictures extracted from digital ureteroscopy with an overall accuracy of 40.7%.^15^ In contrast:, in our study, the overall accuracy of stone recognition for junior urologies was 67.27%. The difference could be due to the use of endoscopic images in the study by Bergot et al., whereas in our study the analysis was done during the endourological procedures.

Recent Judies by Estrade et al. had stones of only one morphological type in 51.40%, while 48.60% were mixed stones, whereas in this current study, pure stones were present in 18.70% of the cases, and mixed stones were in 81.30%.^16^ The overall matching rate was 81.60% in the study by Estrade et al, and our study was 64.44%. A high matching rate was observed in their study between the endoscopic image and microscopic assessment of the stone due to increased urologist: experience for more than 20 years and a biologic of >40 years of experience who observed the stone microscopically. All three layers (the stone surface, stone section, and the nucleus of the stone) of the stone were considered in the study for analysis.^16^ In comparison, our study also showed a high matching rate in the case of senior consultants of 75.00% for pure stones, indicating that increased experience in endoscopic stone management increases the chance of correctly predicting the stone morphology.

Artificial intelligence (AI) for detecting stone composition was analyzed by Black et al. Without any manual image adjustment, the system was able to accurately predict stone type, especially UA (94.00%) and COM (90.00%), with an overall success rate of 85.00% when using a static image.^17^ Similarly, we could predict the Uric acid stones by 84.31%. Future advancements in AI and the use of enhanced and more sophisticated technologies would improve the diagnostic accuracy of the stone morphology.^18-20^

Furthermore, it is important to acknowledge that visual assessment, although valuable, remains inherently subjective and can be influenced by intraoperative factors such as lighting, image resolution, and the presence of blood or debris, which may obscure key morphological features of the stone surface. Studies have also shown that certain mixed stones may present predominantly with the morphological features of one component, leading to potential misclassification if chemical analysis is not performed.^7,16^ Therefore, while morpho-constitutional analysis during endourological procedures provides immediate intraoperative guidance, confirmation with Fourier Transform Infrared Spectroscopy (FTIR) or X-ray diffraction remains the gold standard for definitive diagnosis.^9^

There were a few limitations in our study. The sample of the stone sent for stone analysis may not represent the composition of the whole stone, as the core and outer layer of the stone were not sent separately. The experience level of the surgeon was assessed subjectively with the number of years. A more objective measure, such as the number of endourological procedures performed per week, would have been considered to better quantify surgical expertise.

## CONCLUSION

The overall agreement between visual assessment of urinary stone morphology during endo-urological procedures and Fourier transform infrared spectroscopy-based composition analysis in this study was moderate, with a Cohen’s kappa value of 0.62. This indicates that the level of concordance was substantially higher than chance alone, but not perfect, suggesting that while visual assessment can provide useful intraoperative information, it cannot fully replace formal spectroscopic analysis. Therefore, visual morpho-constitutional assessment can serve as a valuable intraoperative guide and can be used as an adjunct to formal stone analysis by Fourier transform infrared spectroscopy for definitive diagnosis and comprehensive management planning.

## Data Availability

The data are available from the corresponding author upon reasonable request.
